# Pericyte-derived extracellular vesicle-mimetic nanovesicles ameliorate erectile dysfunction via lipocalin 2 in diabetic mice

**DOI:** 10.7150/ijbs.72243

**Published:** 2022-05-16

**Authors:** Limanjaya Anita, Guo Nan Yin, Soon-Sun Hong, Ju-Hee Kang, Yong Song Gho, Jun-Kyu Suh, Ji-Kan Ryu

**Affiliations:** 1 National Research Center for Sexual Medicine and Department of Urology, Inha University College of Medicine, Incheon, 22332, Republic of Korea; 2 Department of Biomedical Sciences, College of Medicine, and Program in Biomedical Science & Engineering, Inha University, Incheon 22332, Republic of Korea; 3 Department of Pharmacology and Medicinal Toxicology Research Center, Inha University College of Medicine, Incheon 22332, Republic of Korea; 4 Department of Life Sciences, Pohang University of Science and Technology, Pohang, Kyeongsangbuk-do 37673, Korea

**Keywords:** Pericyte, Nanovesicles, Lipocalin2, Neurovascular regeneration, Diabetes mellitus, Erectile dysfunction

## Abstract

Diabetes mellitus is one of the main causes of erectile dysfunction (ED). Men with diabetic ED do not respond well to oral phosphodiesterase-5 inhibitors owing to neurovascular dysfunction. Pericyte-derived extracellular vesicle-mimetic nanovesicles (PC-NVs) are known to promote nerve regeneration in a mouse model of cavernous nerve injury. Here, we report that administration of PC-NVs effectively promoted penile angiogenesis and neural regeneration under diabetic conditions, thereby improving erectile function. Specifically, PC-NVs induced endothelial proliferation and migration and reduced cell apoptosis under diabetic conditions. In addition, PC-NVs induced neural regeneration in STZ-induced diabetic mice in dorsal root ganglion and major pelvic ganglion explants *in vivo* and *ex vivo* under high-glucose conditions. We found that lipocalin 2 (Lcn2) is a new target of PC-NVs in this process, demonstrating that PC-NVs exert their angiogenic and nerve-regeneration effects by activating MAP kinase and PI3K/Akt and suppressing P53 signaling pathway in an Lcn2-dependent manner. Our findings provide new conclusive evidence that PC-NVs can promote neurovascular regeneration and recovery of erectile function under diabetic conditions via an Lcn2-dependent mechanism. Thus, local administration of PC-NVs may be a promising treatment strategy for the treatment of diabetic ED.

## Introduction

Erectile dysfunction (ED), defined as incapability to achieve and/or maintain a satisfactory erection during sexual intercourse.^1^, ^2^ ED predicted will affect 322 million men by 2025.^3^, ^4^ It commonly affects after 40 years, increases with age and comorbidities, such as diabetes, hypertension, dyslipidemia, or other hormonal disturbances.^5^, ^6^ Long-term de-vascularization, endothelial 7 and pericyte dysfunction,^8^ smooth muscle cell apoptosis, and peripheral neuropathies results in poor responses to phosphodiesterase-5 (PDE5) inhibitors, the first-line drugs for ED.^9^, ^10^ New targets, such as vascular endothelial growth factor (VEGF),^11^ angiopoietins,^12^ and brain-derived neurotrophic factor (BDNF),^13^ are under investigation. Insufficient efficacy, side effects, and difficulties in protein engineering are the major obstacle of clinical development.^14^, ^15^ Therefore, a highly efficient treatment with low side effects still needed to solve complicated vasculo-neuropathy in diabetic ED.

Pericytes is a vascular mural cell embedded within the basement membrane of micro-vessels and has the ability to differentiate into completely different cell phenotypes.^16^-^19^ Pericytes is important in vasoconstriction, vessel stabilization, and regulation of blood vessel development. We previously reported that pericytes are abundantly distributed in the penis in mice and humans 20 also has potential role in neurovascular regeneration in mouse models of diabetic ED and cavernous nerve injury-induced ED.^21^, ^22^ However, little is known about the detailed functions and molecular mechanisms by which pericytes promote penile neurovascular regeneration under pathologic conditions.

Extracellular vesicles (EVs) secreted to the extracellular space by cell as micro-vesicles, exosomes, and apoptotic bodies.^23^, ^24^ EVs consist of lipids, proteins and nucleic acids that involved in physiological and pathological intercell communication.^25^, ^26^ Given their release pathways, size, and contents, EVs gain extensive attention as new drug-delivery system.^27^ EVs derived from specific cell types has tissue regeneration effects.^28^, ^29^ However, low yield is a hurdle for the EVs production. To overcome the limitation, cell-extruder system employed to extract 100-fold greater amounts of EV-mimetic nanovesicles (NVs) from different cell types, such as embryonic stem cells (ESC-NVs) and pericytes (PC-NVs), that has similar characteristic to those of natural EVs.^22^, ^30^, ^31^ Nevertheless, detailed mechanisms by which PC-NVs promote penile neurovascular regeneration in diabetic ED have not yet been explored.

This verifies erectile function enhancement of exogenic PC-NVs treatment reinstating cavernous mural and endothelial cell content; endothelial NOS (eNOS) phosphorylation, endothelial tight junction integrity; reducing cavernous vessels permeability; and promote nerve regeneration. Furthermore, angiogenic (MCECs migration and tube formation, aortic ring sprouting, and proliferation assays) and neurogenic (dorsal root ganglion and major pelvic ganglion neurite sprouting assays) effect of PC-NVs under high-glucose conditions. Additionally, RNA sequencing and Signaling Explorer Antibody Arrays analyses revealed that PC-NVs promote penile neurovascular regeneration under diabetic conditions through lipocalin-2 (Lcn2)-dependent stimulating mitogen-activated protein kinase (MAPK), phosphoinositide 3-kinase (PI3K)/Akt signaling pathways and suppress the tumor protein 53 (Trp53) signaling pathway.

## Results

### Characterization of PC-NVs

**Figure 1:**
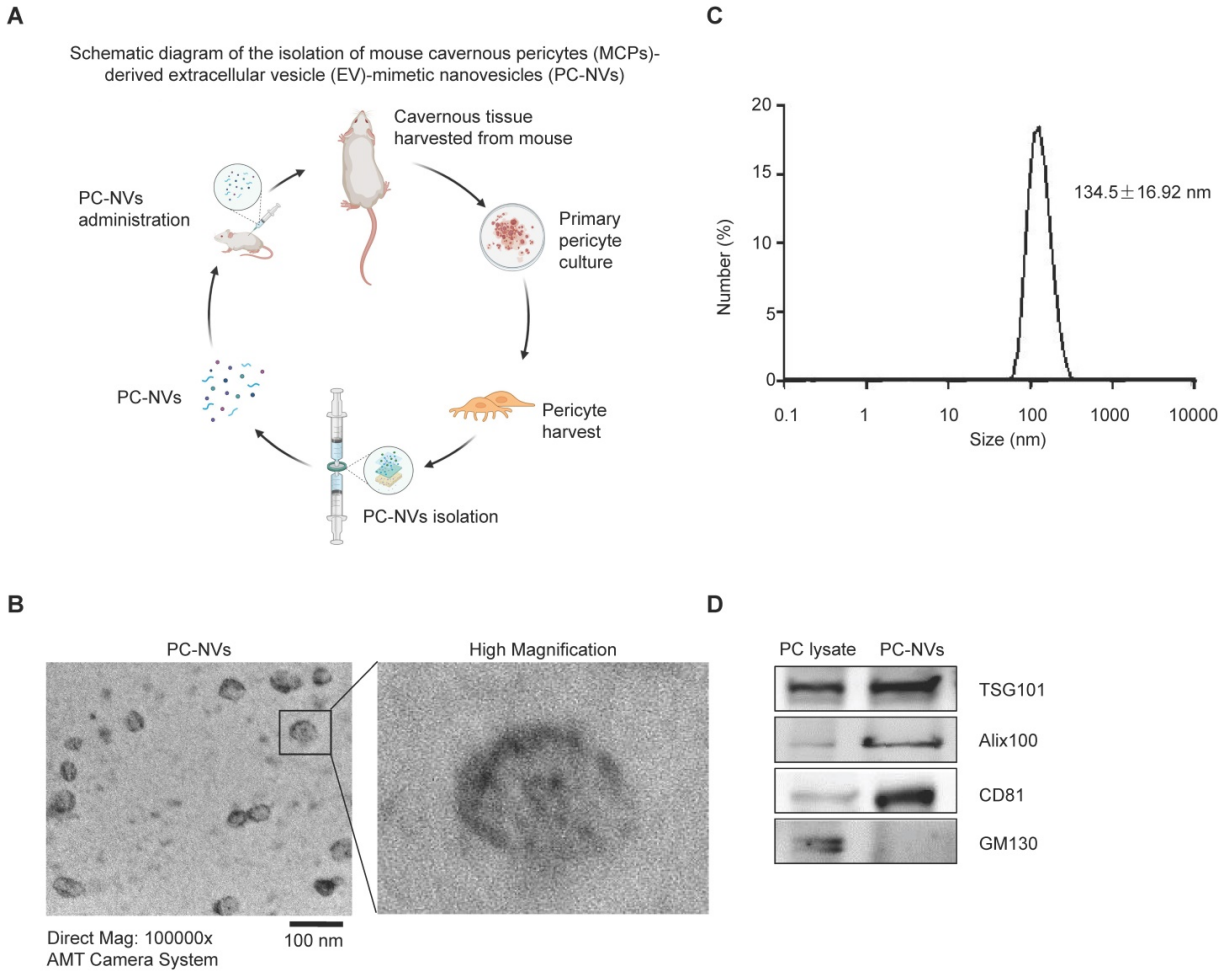
** Preparation and characterization of MCP-derived PC-NVs.** (A) Schematic overview of PC-NVs extraction procedure. (B) Representative TEM phase images for detecting isolated PC-NVs. Scale bar = 100 nm. (C) Enrichment diameter distribution of PC-NVs, measured by dynamic light scattering. (D) Representative Western blot analysis of EV markers from three independent experiments that yielded similar results. GM130: negative PV marker; TSG101, Alix100 and CD81: positive EV markers. HBS, HEPES-buffered saline.

PC-NVs were extracted from mouse cavernous pericytes accordingly^30^, ^32^ (see **Figure 1**A), characterized using Zetasizer Nano ZS analysis, transmission electron microscopy (TEM), and Western blotting. Zetasizer Nano ZS analysis (**Figure 1**B) and TEM images (**Figure 1**C) revealed vesicular cup-like structure of PC-NVs with enrichment diameter of 134.5 ± 16.92 nm. Characteristic and similarities of PC-NVs to the natural EV were summarized at **Table S1.** Western blot confirmed positive EV surface markers, TSG101, Alix, and CD81, were present in PC-NVs but not in lysates, whereas the negative EV surface marker GM130 was not presented in purified PC-NVs (**Figure 1**D).

### Tracking analysis of PC-NVs in the mouse penis

To determine the distribution of PC-NVs, DiD a red-dye labeled PC-NVs was injected into the mouse penis and harvested at the indicated timepoints for tracking analysis of PC-NVs by confocal microscopy. Representative images indicate DiD-labeled PC-NVs (red) were present and co-localized with the endothelial cell marker PECAM-1 (green) in corpus cavernosum tissue up to 24 hours (**Figure S1**).

### Intracavernous injection of PC-NVs improves erectile function in diabetic mice

**Figure 2:**
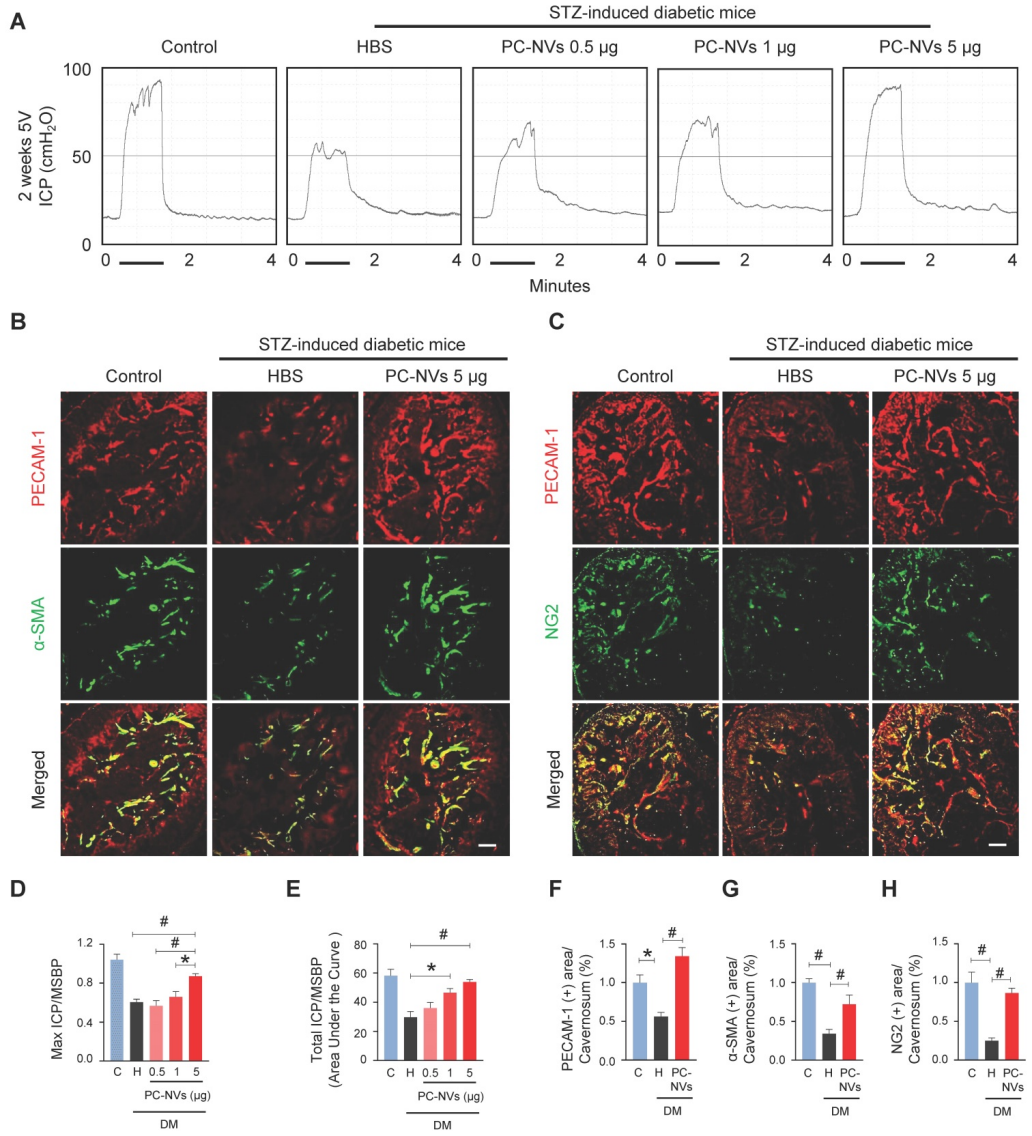
** PC-NVs improve erectile function in diabetic mice.** (A) Representative ICP responses for age-matched control (C) and diabetic mice 2 weeks after intracavernous injection of HBS (H) or PC-NVs (0.5, 1, and 5 μg/20 μl) on days -3 and 0. Stimulus interval indicated by a solid bar. (B and C) Staining for endothelial cells (PECAM-1; red), smooth muscle cells (α-SMA; green), and pericytes (NG2; green) of cavernous from age-matched control (C) and diabetic mice 2 weeks after intracavernous injection of HBS (H) or PC-NVs (5 μg/20 μl) on days -3 and 0. Scale bar = 100 μm. (D and E) Ratios of mean maximal ICP and total ICP (area under the curve) to MSBP calculated for each group and presented as means ± SEM (n = 5). (F-H) Quantitative analysis of cavernous endothelial cell, smooth muscle cell and pericyte content, quantified by Image J and presented as means ± SEM (n = 7; *P < 0.05; ^#^*P* < 0.001). DM, diabetes mellitus; HBS, HEPES-buffered saline; STZ, streptozotocin.

Pericytes known as regulator of vascular stability and regeneration.^33^, ^34^ Potential effect of PC-NVs was measured with erectile function study with electrical stimulation of cavernous nerve (5V, 12Hz, and 1m/s) 2 weeks after intracavernous injection of HBS or different amounts of PC-NVs (0.5, 1, or 5 µg in 20 μl of HBS) on days -3 and 0 in age-matched control and diabetic mice (**Figure 2**A). Ratio of maximal and total intracavernous pressure (ICP) to mean systolic blood pressure (MSBP) significantly declined in HBS-treated diabetic mice compared with age-matched control. Marked improvement shown by diabetic mice after two high dose intracavernous injections of PC-NVs (5 µg/20 µL) to 85% of those in controls. Partially improved erection parameters observed in lower doses (0.5 and 1 µg per 20 µL), (**Figure 2**A, **2**D and **2**E). No significant MSBP variations were found between experimental and control groups. Immunofluorescence staining for PECAM-1 (endothelial cell marker), α-SMA (smooth muscle cell marker), and NG2 (pericyte marker) in cavernosum tissue demonstrated enhancement of endothelial cell, smooth muscle cell, and pericyte content in diabetic mice after PC-NVs injections (**Figure 2**B, **2**C and **2**F-**2**H). Treatment with PC-NVs (days -3 and 0; 5 µg in 20 μl of HBS) significantly improved levels of p-eNOS (**Figure S2**), expression of the tight junction proteins claudin-5 and occludin in diabetic mice (**Figure S3A-S3C**), suppressed endothelial permeability (extravasation of oxidized-LDL; **Figure S3D and S3E**). Fasting and postprandial blood glucose concentrations significantly higher in diabetic than those in control mice. Regardless of treatment no significant differences between body weight and blood glucose levels of diabetic mice (**Table S2**) suggesting that PC-NVs restored cavernous endothelial-mural cell content and ameliorate erectile dysfunction in diabetic mice.

### PC-NVs induce angiogenesis by increasing proliferation and reducing apoptosis of cavernous endothelial cells under high-glucose conditions

**Figure 3:**
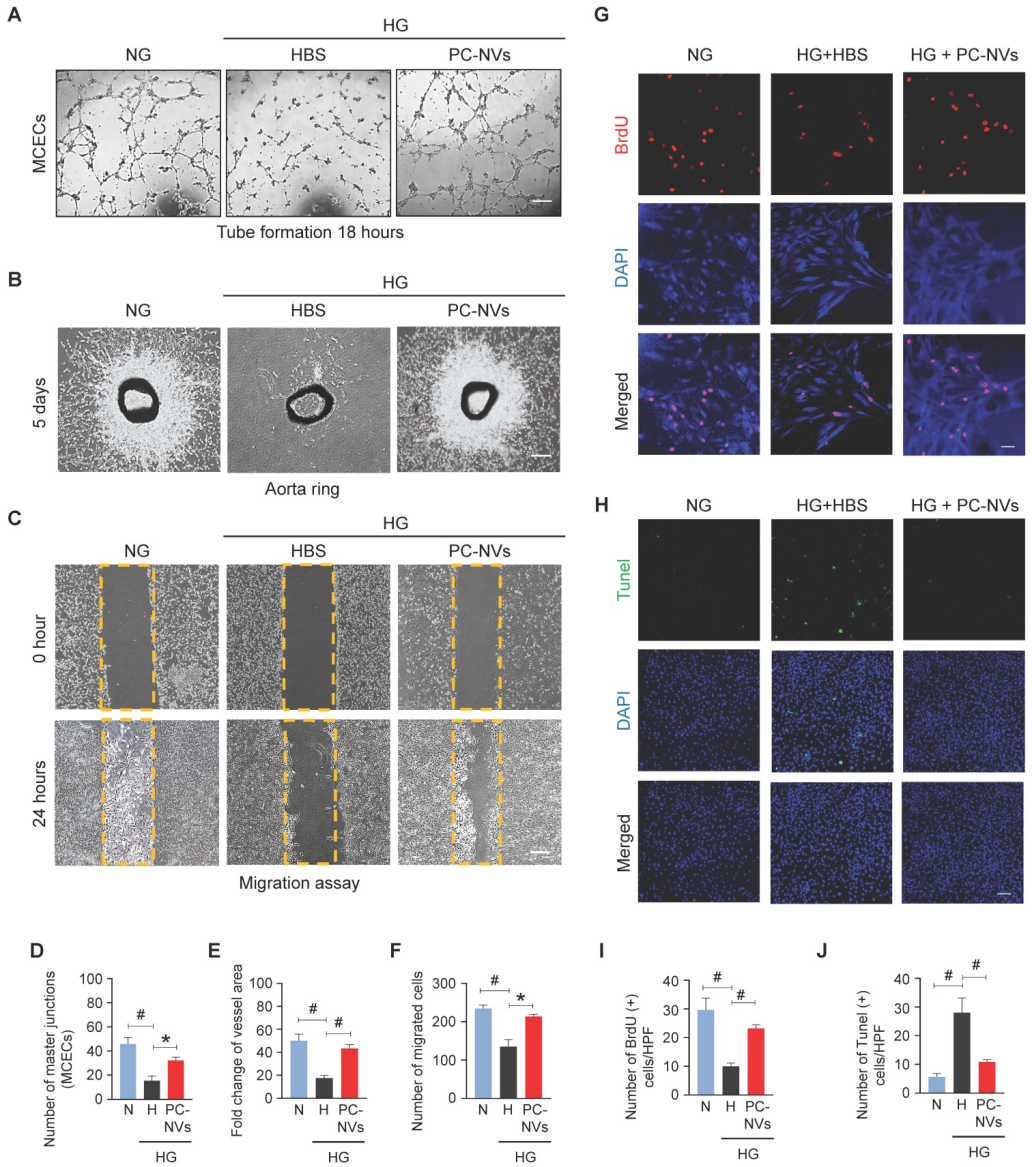
** PC-NVs enhance angiogenesis by increasing proliferation and decreasing apoptosis of MCECs under HG conditions.** (A) Tube-formation assay. MCECs were treated with PC-NVs (1 µg/mL) or HBS under NG or HG conditions for 3 days. Representative images were acquired at 18 hours. Scale bar = 100 µm. (B) *Ex vivo* mouse aortic ring microvessel-outgrowth assay. Aorta rings were treated with PC-NVs (1 µg/mL) or HBS under NG or HG conditions for 5 days. Representative images of sprouting microvessels were acquired at 5 days. Scale bar = 100 µm. (C) Migration assay. MCECs were treated with PC-NVs (1 µg/mL) or HBS under NG or HG conditions for 3 days. Representative images of migrated cells were acquired at 24 hours. Scale bar = 100 µm. (D) Master junctions, quantified using Image J and presented as means ± SEM (n= 5). (E) Intensity of microvessel sprouting area from aortic rings, quantified using Image J and presented as means ± SEM (n = 9). Relative ratio of the NG group was defined as 1. (F) Migrated cells, quantified using Image J and presented as means ± SEM (n = 5). (G and H) Immunofluorescence staining of MCECs with anti-BrdU antibody (red) (G) and TUNEL assay (H) in cells treated with PC-NVs (1 µg/mL) or HBS under NG or HG conditions for 3 days. Nuclei were labeled with DAPI. Scale bars = 50 μm. (I and J) Number of BrdU-positive (I) or TUNEL-positive (J) endothelial cells per high-power field (HPF). Results are presented as means ± SEM (n = 5; *P < 0.05; ^#^*P* < 0.001). DAPI, 4,6-diamidino-2-phenylindole; TUNEL, terminal deoxynucleotidyl transferase-mediated deoxyuridine triphosphate nick-end labeling.

In diabetes, endothelial dysfunction is a reflection of apoptosis, diminished proliferation and migration.^35^ Mouse cavernous endothelial cells (MCECs) revealed declines of endothelial tubes formed under high-glucose (HG; 30 mM) conditions and HBS (**Figure 3**A). To determine PC-NVs pro-angiogenic activity we performed aortic ring microvessel sprouting assays (**Figure 3**B) and migration assays (**Figure 3**C) under normal-glucose (NG; 5 mM) and HG conditions. Comparably MCECs tube formation assays, aortic ring microvessel-sprouting activity, and endothelial cell migration were significantly reduced under HG conditions (with HBS). HG-related discrepancies were completely rescued by PC-NVs treatment (**Figure 3**A-**3**F). To understand how PC-NVs induce angiogenesis, we evaluated mouse endothelial cell proliferation (BrdU incorporation assay; **Figure 3**G) and apoptosis (TUNEL assay; **Figure 3**H) under HG conditions. Diminished proliferation and augmented apoptosis of MCECs under HG conditions (with HBS); restored to baseline values after addition of PC-NVs (**Figure 3**G-**3**J). Taken together, these suggest that PC-NVs induce angiogenesis under HG conditions by promoting endothelial cells migration and proliferation also inhibiting cell apoptosis.

### PC-NVs preserve neurotrophin function under diabetic conditions

**Figure 4:**
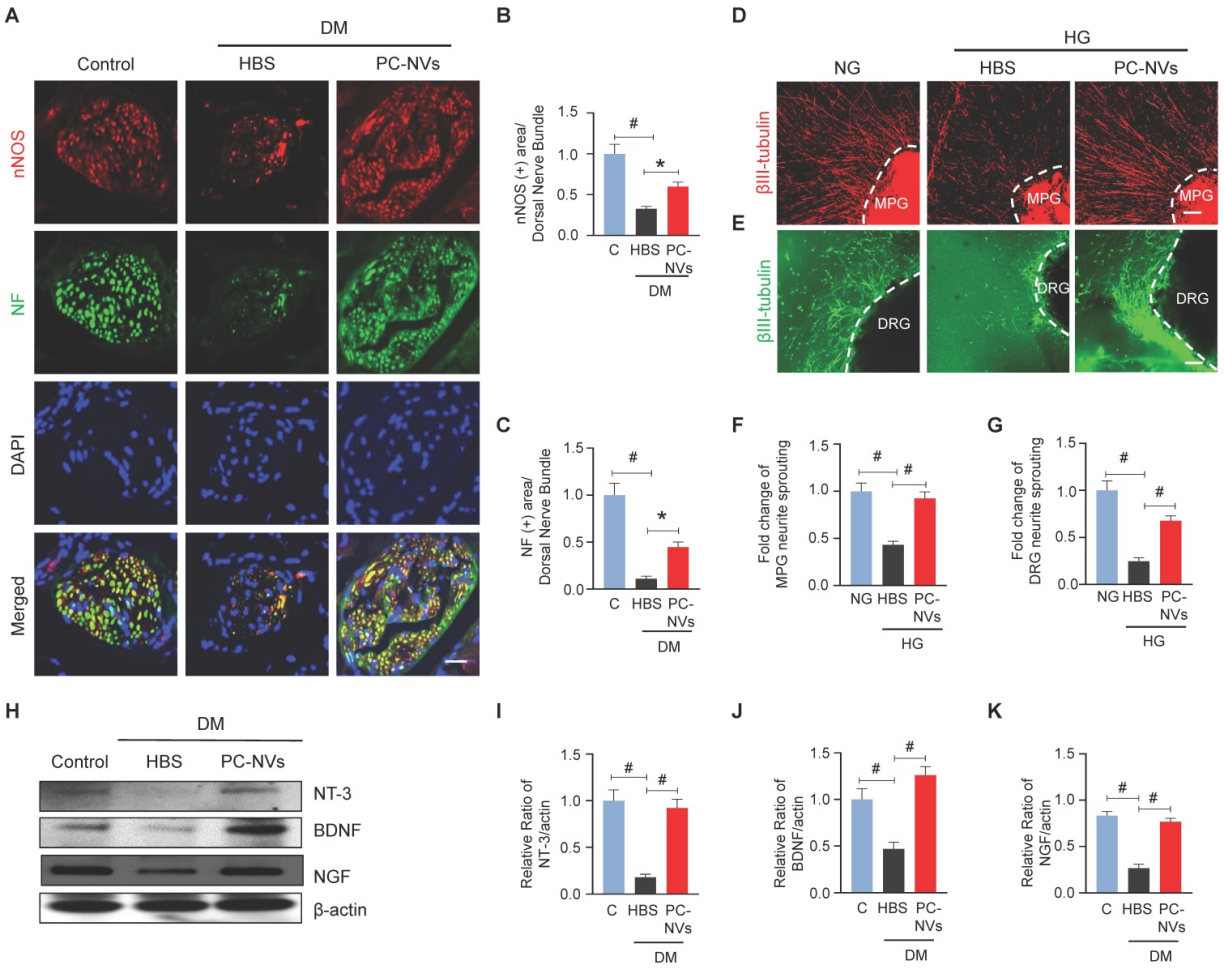
** PC-NVs induce neural regeneration under diabetic or HG conditions.** (A) nNOS (red) and NF (green) staining in cavernous tissue from age-matched control (C) and diabetic mice 2 weeks after intracavernous injection of HBS or PC-NVs (5 μg/20 μl) on days -3 and 0. Nuclei were labeled with DAPI. Scale bar = 25μm. (B and C) Quantitative analysis of nNOS- and NF-positive axonal areas by ImageJ, presented as means ± SEM (n = 6). (D and E) βIII-tubulin staining in mouse MPG (red) and DRG (green) tissue treated with PC-NVs (1 µg/mL) or HBS under NG or HG conditions for 5 days. Scale bar = 100 μm. (F and G) βIII-tubulin-immunopositive neurite length in MPG or DRG tissue, quantified using Image J and presented as means ± SEM (n = 7). (H) Representative Western blots for neurotrophic factors (NGF, NT3, and BDNF) in cavernous tissue from age-matched control (C) and diabetic mice 2 weeks after intracavernous injection of HBS or PC-NVs (5 μg/20 μl) on days -3 and 0. (I-K) Band intensity values of each neurotropic factor normalized to the density of β-actin, quantified using Image J and presented as means ± SEM (n = 6; *P < 0.05; ^#^*P* < 0.001). The relative ratio in the control group was defined as 1. DM, diabetes mellitus; HBS, HEPES-buffered saline.

In diabetic related ED, neuropathy is one of contributory factor.^36^ Our studies disclosed the significancy of PC-NVs induced neurovascular regeneration on CNI-model.^22^ To validate that PC-NVs impacting neuronal regeneration in diabetic, we evaluated neuronal NOS (nNOS) and neurofilament-2000 (NF) expression on mouse cavernous dorsal nerve bundles. Interestingly, diminished nNOS and NF expression in HBS-treated diabetic mice revived by PC-NVs treatment (**Figure 4**A-**4**C). We sought to determine the effect of PC-NVs on neurite outgrowth in MPG and DRG explants under HG conditions. MPG and DRG immunofluorescence staining for βIII-tubulin revealed greatly reduced neurite sprouts under HG condition (with HBS); furthermore, PC-NVs exerted a rescue effect, profoundly enhancing neurite outgrowth under HG (**Figure 4**D-4G). To rule whether the effects of PC-NVs on neuronal regeneration are mediated by production of neurotrophic factors, neurotrophin-3 (NT-3), brain-derived neurotrophic factor (BDNF), and nerve growth factor (NGF) expression, mouse cavernous tissues were evaluated by Western blotting. Particularly, these neurotrophic factors expression in diabetic mice received PC-NVs were greater compared to those received HBS-injection **(Figure 4**H-**4**K). These results suggest that PC-NVs promote nerve regeneration under diabetic conditions by increasing the expression of NT-3, BDNF, and NGF.

### Lcn2 is a novel target in PC-NV-triggered neurovascular regeneration under diabetic conditions

**Figure 5:**
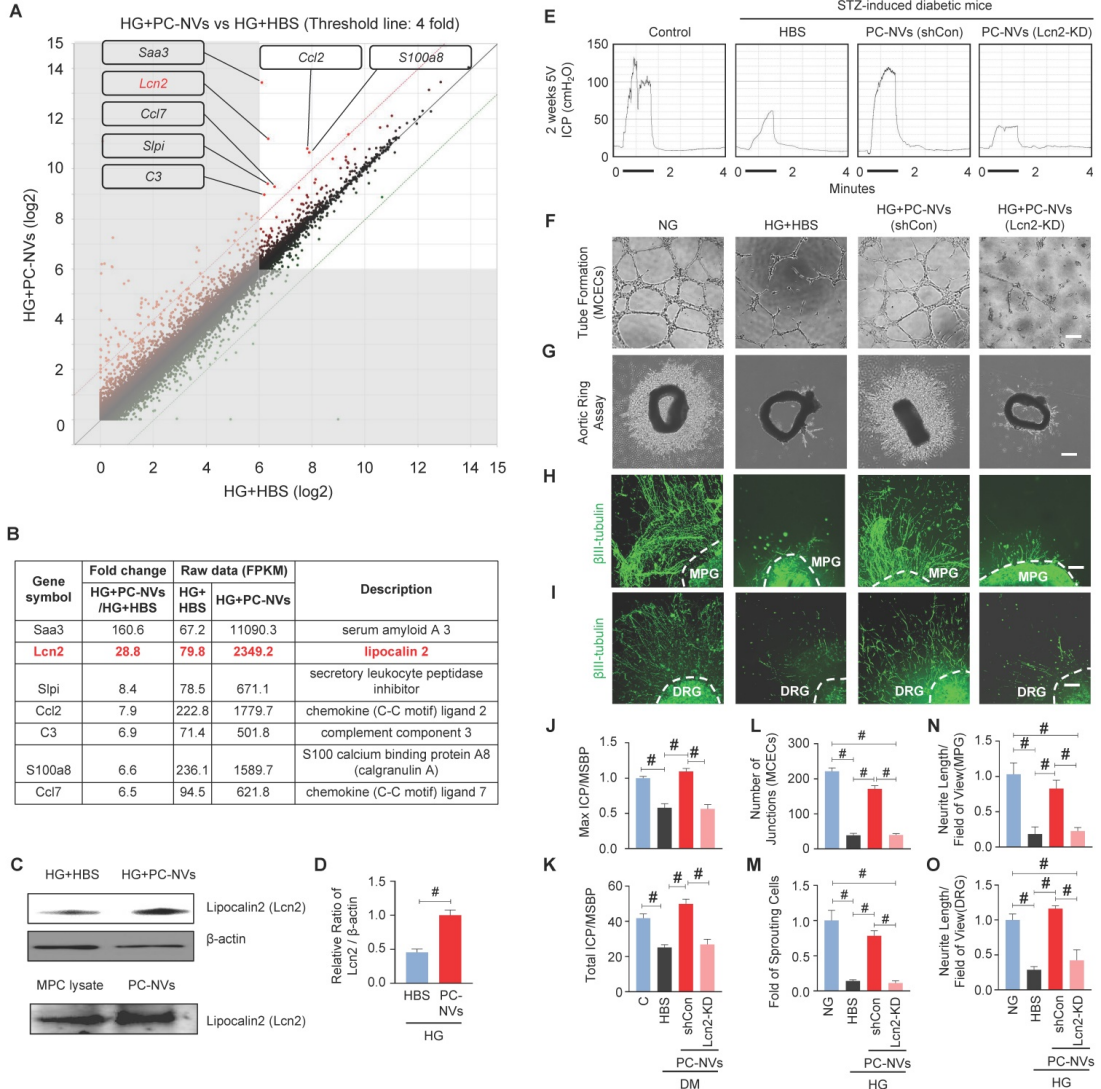
** Lcn2 as a novel target of PC-NV-triggered neurovascular regeneration under diabetic conditions.** (A and B) Analysis of RNA-sequencing data from MCECs treated with PC-NVs (1 µg/mL) or HBS under HG conditions for 3 days. (A) DEGs between HG+HBS and HG+PC-NVs, shown by scatter plot. (B). Seven genes met criteria for significant DEGs (fold change > 4 and log2 > 6); Lcn2 (red) identified as a target for PC-NV-induced neurovascular regeneration. (C and D) Lcn2 Relative expression in MCECs treated with PC-NVs (1 µg/mL) or HBS under HG conditions in MCP lysates (C, top) and PC-NVs (C, bottom), detected by Western blotting. (D) Band intensity of Lcn2 normalized to that of β-actin, quantified using Image J and presented as means ± SEM (n = 5). (E) Representative ICP responses for age-matched control (C) and diabetic mice obtained 2 weeks after intracavernous injection of HBS (H), PC-NVs (shCon) (5 μg/20 μl), or PC-NVs (Lcn2-KD) (5 μg/20 μl) on days -3 and 0. The stimulus interval indicated by a solid bar. (F) Tube-formation assay in MCECs treated with HBS, PC-NVs (shCon), or PC-NVs (Lcn2-KD) under NG or HG conditions for 3 days. (G) *Ex vivo* mouse aortic ring microvessel-outgrowth assay. Aorta rings were treated with HBS, PC-NVs (shCon), or PC-NVs (Lcn2-KD) under NG or HG conditions for 5 days. (H and I) βIII-tubulin staining in mouse MPG (H) and DRG (I) tissue, treated with HBS, PC-NVs (shCon), or PC-NVs (Lcn2-KD) under NG or HG conditions for 5 days. (J and K) Ratios of mean maximal ICP and total ICP (area under the curve) to MSBP, calculated for each group and presented as means ± SEM (n = 5). (L) Master junctions, quantified using Image J and presented as means ± SEM (n = 9). (M) Intensity of microvessel sprouting area from aortic rings, quantified using Image J and presented as means ± SEM (n = 5). (N and O) βIII-tubulin-immunopositive neurite length in MPG or DRG tissue, quantified using Image J and presented as means ± SEM (n = 4; ^#^*P* < 0.001). DM, diabetes mellitus; HBS, HEPES-buffered saline.

Extracellular vesicles are known to carry genetic components (mRNAs and miRNAs), lipids, and numerous proteins, which important in physiological and pathological communication between cells.^25^, ^26^ To clarify how PC-NVs mediating neurovascular regeneration, we performed RNA sequencing analyses of MCECs under HG conditions with HBS or PC-NVs treatment. There were 23183 genes detected in two libraries. Seven qualifying genes determined as significant in Differentially Expressed Genes (DEGs) with fold-change > 4 and log2 > 6 (**Figure 5**A and **5**B). All seven genes were increased in HG conditions with PC-NVs treatment (HG+PC-NVs) compared with HG+HBS treatment. We assumed that PC-NVs carried and transfer these genes, that directly or indirectly affect neurovascular regeneration under HG conditions. A literature review identified Lcn2 has the closest relationship with neurovascular regeneration. Consistent RNA sequencing result, the expression of Lcn2 protein was significantly increased in MCECs treated with HG+PC-NVs compared with those treated with HG+HBS (**Figure 5**C, top and **5**D). Remarkably, Lcn2 protein was also detected in pericyte cell lysates and PC-NVs (**Figure 5**C, bottom). To evaluate whether Lcn2 protein is responsible for PC-NV-induced neurovascular regeneration under diabetic conditions, we knockdown Lcn2 expression in mouse cavernous pericytes (MCPs) by infection with lentivirus expressing small hairpin RNA (shRNA) targeting Lcn2 (shLcn2) or scrambled shRNA control (shCon) lentivirus, and isolated Lcn2-KD PC-NVs for *in vitro* studies (**Figure S4**). Erectile function was assessed by electrical stimulation of the cavernous nerve after injection of Lcn2-KD PC-NVs as described in Materials and Methods. Maximal and total intracavernous pressure (ICP) relative to MSBP were significantly improved in diabetic mice treated with shCon PC-NVs (5 µg/20 µL). However, treatment with shLcn2-KD PC-NVs had no effects in diabetic mice (**Figure 5**E, **5**J and **5**K). No detectable differences in MSBP were found between experimental groups (**Table S3**). Similar results were observed in MCEC tube-formation assays (**Figure 5**F and **5**L), aortic ring microvessel sprouting assays (**Figure 5**G and **5**M), and MPG (**Figure 5**H and **5**N) and DRG (**Figure 5**I and **5**O) neurite outgrowth assays. These results suggest that Lcn2 in PC-NVs plays an important role in promoting neurovascular regeneration in diabetic conditions.

### Identification of the PC-NVs/Lcn2 signaling pathway responsible for promoting neurovascular regeneration

**Figure 6:**
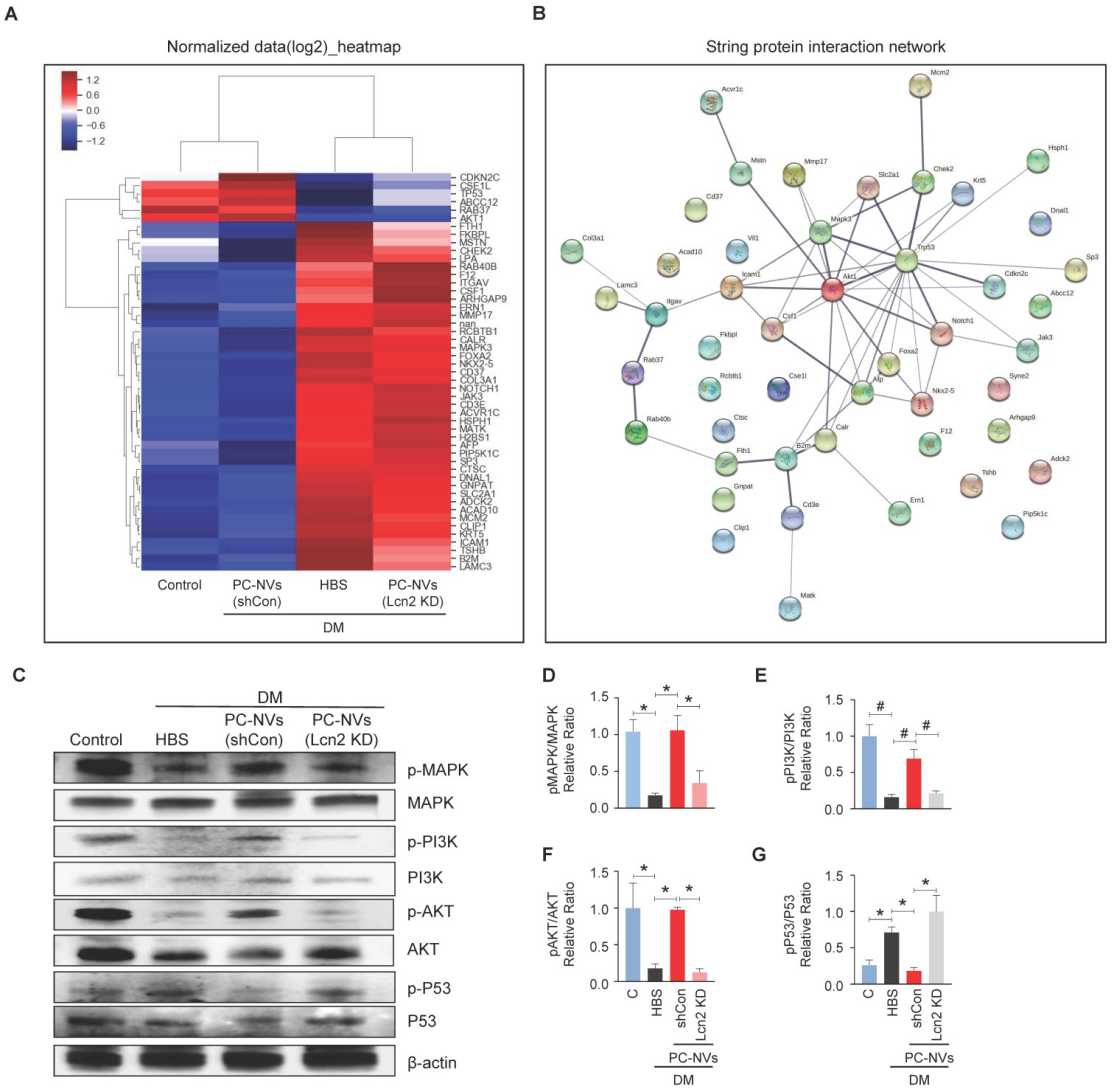
** Identification of the PC-NVs/Lcn2 signaling pathway responsible for promoting neurovascular regeneration.** (A) Heatmap of log2-normalized intensities of DEGs from age-matched controls (C) and diabetic mice, determined 2 weeks after intracavernous injections of HBS, shCon-PC-NVs (5 μg/20 μl) or Lcn2-KD-PC-NVs (5 μg/20 μl) on days -3 and 0. Color density expressed fold-change magnitude in the indicated multiples. Red, up-regulated; blue, down-regulated. (B) Protein-protein interaction network of DEGs. The line thickness corresponds to the interaction strength between protein (C) Representative Western blots for MAPK, PI3K, Akt, and Trp53 in cavernous tissue from age-matched control (C) and diabetic mice, 2 weeks after intracavernous injections of HBS, shCon-PC-NVs (5 μg/20 μl) or Lcn2-KD-PC-NVs (5 μg/20 μl) on days -3 and 0. (D-G) Band intensities of each protein normalized to the density of β-actin, quantified using Image J and presented as means ± SEM (n = 4; *P < 0.05; ^#^*P* < 0.001). DM, diabetes mellitus; HBS, HEPES-buffered saline.

To elucidate the PC-NV/Lcn2-mediated signaling pathway, we profiled mouse cavernosum tissues from age-matched control and diabetic mice received HBS (H), PC-NVs, or Lcn2-KD PC-NVs with Signaling Explorer Antibody Arrays, which employ 1358 unique antibodies covering 20 cell signaling pathways. Out of 1358 protein targets, only 49 contra-regulated proteins showed increases or decreases in DM/Control and Lcn2-KD PC-NVs/shCon PC-NVs expression ratios greater than 1.2 (**Figure 6**A). STRING database were used to analyze the protein-protein interaction (PPI) network, integrates known and predicted PPIs to predict protein functional interactions.^37^, ^38^ PPI network analysis identified MAPK3, Akt1, and Trp53 as key proteins regulated by PC-NVs in Lcn2-dependent manner under diabetic conditions (**Figure 6**B). Further validation studies exhibited that the neurovascular regeneration induced by PC-NVs under diabetic conditions was achieved by activating MAPK, PI3K/Akt signaling, and suppressing the Trp53 pathway in an Lcn2-dependent manner (**Figure 6**C-**6**G). Taken together, these findings suggest that MAPK, Akt, and Trp53 signaling pathways constitute the key pathways for PC-NV/Lcn2-mediated neurovascular regeneration under diabetic conditions.

## Discussion

Chronic hyperglycemia associated change of vascular endothelial, smooth muscle, pericytes, and neuronal cells in penile tissue.^20^, ^39^, ^40^ According to literature, pericytes are involved at the early angiogenesis and vasculogenesis.^41^ We recently demonstrated that PC-NVs promote nerve regeneration by enhancing cell survival signaling and neurotrophic factors of cavernous nerve injury (CNI) induced ED.^22^ For the first time we demonstrated that PC-NVs promote penile neurovascular regeneration in diabetic mice in an Lcn2-dependent manner. **Figure S5** summarize the detailed mechanisms of action of exogenous PC-NVs in improving diabetic ED.

Emerging evidence has shown that EVs are not merely excreted cellular waste or by-products, but also a nano carrier-transport, delivering bioactive intra-cell materials to target cells and regulates biological activity of the recipient cells.^42^ Pericytes shares mesenchymal stem cells characteristics and constitute as vital part of the neurovascular unit.^43^ Pericyte loss is considered an early sign of diabetes-related microvascular diseases, such in retinopathy and nephropathy.^44^ despite of its benefit in neurovascular regeneration of CNI-ED model, no published data examined the use of PC-NVs for diabetic ED.^22^ Accordingly, we hypothesized that PC-NVs can exert beneficial effects in diabetic ED. To test our hypothesis, we exogenously injected PC-NVs into penis tissues of STZ-induced diabetic mice. Subsequent analyses reveal that PC-NVs increased endothelial cell and mural cell content (smooth muscle cells and pericytes), p-eNOS levels, expression of endothelial cell-cell junction proteins, MCEC migration and tube formation, aortic ring sprouting, expression of neurotrophins (NT-3, BDNF, and NGF) and neurite sprouting in MPG and DRG tissues. These effects synergistically salvaged neuro-vasculopathies in STZ-induced diabetic mice and finally improved erectile function. These findings suggest that local injection of PC-NVs may be a promising strategy for the treatment of diabetic ED.

RNA sequencing technology were used to analyzed constantly changing cellular transcriptome and identify Lcn2 a novel target for PC-NVs. Lcn2 known as a pro-inflammatory protein in the central nervous system and has important roles in many organ systems.^45^ Lcn2 binds and regulates matrix metalloproteinases likewise their inhibitors, thereby affecting neurovascular damage and remodeling.^46^ Recent studies shown Lcn2 exerts different effects at different concentrations. It may promote neuronal cell death at high concentrations, recruiting microglia and astrocytes to protect nerves, otherwise released by damaged neurons as a “help” signal under certain conditions.^47^ Lcn2 also act as an angiogenic factor, by promoting neurovascular remodeling.^48^, ^49^ Current study proved that Lcn2-depleted PC-NVs cannot drive neurovascular regeneration or restore erectile function under diabetic conditions. Interestingly, the Lcn2 not only highly expressed in PC-NVs, but also in MCECs after PC-NVs treatment under HG conditions. Suggesting that PC-NVs may act as intercellular delivery vehicles for transfer of Lcn2 to recipient cells and tissues, thus promote neurovascular regeneration under diabetic conditions. However, additional studies needed to prove the augmented expression of Lcn2 in MCECs is attributable to direct transfer from PC-NVs rather than regulation by other pathways.

It has been reported that PC-NVs improved erectile function by enhancing cell survival signaling and expression of neurotrophic factors in a mouse CNI model.^22^ In addition, our previous studies showed that embryonic stem cell-derived EV-mimetic NVs can also restore erectile function in a mouse diabetes model by inducing cell proliferation and survival signaling pathways.^30^ Consistently, Signaling Explorer Antibody Arrays demonstrated that MAPK3, Akt1, and Trp53 signaling constitute the significant signaling pathways for PC-NVs/Lcn2-driven neurovascular regeneration in DM. Further research is needed to confirm that these signaling pathways also operate in neuronal cells under diabetic conditions and confirm if Lcn2 directly secreted or bind to the receptor to activate and regulate these signaling pathways. Knockdown of Lcn2 may also alter another pathway associated with the erectile function declines, and further studies may need to demonstrate whether the same cascade applied after lcn2 knockdown, and whether the same pathway also applies to neuronal cell recovery in diabetic conditions.

In summary, both of PC-NVs *in vitro* and *in vivo* models identified a potential cell signaling network of PC-NVs, providing new insight the benefits of PC-NVs in diabetic conditions. Notably, the exogenous PC-NVs rescue damaged erectile tissues through Lcn2-dependent activation of MAP kinase, PI3K/Akt pathways and suppress the Trp53 signaling pathways, thereby enhancing neurovascular regeneration. Local injection of PC-NVs may attest a paradigm shift for the development of new therapies, not only for ED, but also for other vascular and/or neurological diseases.

## Materials and Methods

### Ethics Statement and Animal Study Design

Eight-week-old male C57BL/6J mice (Orient Bio, Seongnam-si, Gyeonggi-do, Korea) were used in this study. All animal experiments were performed in compliance with Institutional Animal Care and Use Committee of Inha University (approval number: 200309-691). In this study, total of 150 adult male C57BL/6J mice were used: 30 for mouse cavernous pericytes (MCPs) primary culture and pericyte-derived EV-mimetic NV isolation; 30 for diabetes mouse model preparation and related experiments; 20 for mouse cavernous endothelial cell (MCEC) primary culture and aortic ring sprouting assays; 20 for *ex vivo* dorsal root ganglion (DRG) and major pelvic ganglion (MPG) neurite sprouting assays; 50 for RNA sequencing and other signaling pathway studies. All experiments were conducted in blinded manner.

### Mouse Cavernous Pericyte Culture

Primary MCPs were prepared from 8-weeks male C57BL/6J mouse penile 20, 50. The urethra, gland penis, and dorsal nerve bundle were removed and the remaining cavernous tissue submerged in Hank's balance salt solution (Gibco, Carlsbad, CA, USA). Explants were washed two times with PBS and dissected into 1-mm-sized pieces and placed on pre-collagen-I coated 35-mm plates (BD Biosciences, Bedford, MA, USA). Tissues were allowed to set at the bottom of the plate by gravity for 30 minutes with 300 µl of complement Dulbecco's modified Eagle Medium (DMEM, Gibco, Carlsbad, CA, USA) containing 20% fetal bovine serum (FBS; Gibco), 1% penicillin/streptomycin (Gibco), and 10 nM human pigment epithelium-derived factor (PEDF; Sigma-Aldrich, St Louis, MO, USA). Additional 900 µl of the complement media added to the plate before further incubation at 37°C with 5% CO2. The medium was changed every 2 days, and sub-cultured after 10 days. Cell type was determined by staining the cells with pericyte markers; PDGFR-β, NG2 chondroitin sulfate proteoglycan, endothelial cell marker (CD31), smooth muscle cell marker (smooth muscle α-actin), or fibroblast marker (CD90). Human brain microvascular pericytes (HBMP) were used as positive control. Rat aorta smooth muscle cell line (A7r5) and mouse embryonic fibroblast cell line (NIH3T3) was used as negative controls. Cells from passages 2 to 4 were used for all experiments.

### Mouse Cavernous Endothelial Cell Culture

Primary MCECs were prepared from mouse penis tissues as previously described 12 and cultured in 3 mL of complement Medium 199 (M199; Gibco) containing 20% FBS (Gibco), 1% penicillin/streptomycin (Gibco), 0.5 mg/mL heparin (Sigma-Aldrich), and 5 ng/mL VEGF (R&D Systems Inc., Minneapolis, MN, USA). Only the sprouting cells obtained after 2 weeks were used for subcultivation. Cells from passages 2 to 4 were used for all experiments.

### Extraction and Identification of MCP-derived EV-mimetic NVs

MCP-derived EV-mimetic NVs (PC-NVs) were prepared using a mini extruder system (Avanti Polar Lipids, Birmingham, AL, USA) according to the procedure in our previous study^30^, ^32^ and in **Figure 1**. Three 100 mm dishes (each dish containing approximately 5 x 10^6^ MCPs ) were washed 3 times with phosphate-buffered saline (PBS; Gibco), detached with 0.25% trypsin-EDTA (Gibco), and re-suspended in 4-(2-hydroxyethyl) piperazine-1-ethanesulfonic acid (HEPES)-buffered saline (HBS; Gibco). The cell suspension was sequentially extruded 10 times for each pore-sized polycarbonate membranes (10-, 5-, and 1-μm) (Nucleopore; Whatman Inc., Clifton, NJ, USA) and subjected to ultracentrifugation at 100,000 × g for 2 hours at 4°C using step gradient of 50% iodixanol (1 mL; Axis-Shield PoC AS, Oslo, Norway), overlaid with 10% iodixanol (2 mL), lastly topped with the extruded samples (7 mL). Pellets resuspended in HBS and filtered with 0.45-μm filter before stored at -80°C until further use. PC-NVs were quantified with EXOCET exosome quantitation assay kit (System Biosciences, Palo Alto, CA, USA), with adjusted concentration 1 µg/µL. Extracted PC-NVs enrichment diameter was measured by dynamic light scattering using a Zetasizer Nano ZS instrument (Malvern Instrument Ltd., Malvern, UK) and the morphology was assessed by transmission electron microscopy (TEM; Electron Microscopy Sciences, Fort Washington, PA, USA).^30^, ^32^ Extracted PC-NVs and MCP cell lysates were separated by sodium dodecyl sulfate-polyacrylamide gel electrophoresis (SDS-PAGE), probed with antibodies to the negative EV marker GM130 (1:500; BD Biosciences, San Jose, CA, USA) and positive EV markers Alix100 (1:500; Novus Biologicals, Littleton, CO,USA), CD81 (1:500; Novus Biologicals), and TSG101 (1:500; Novus Biologicals).

### Fluorescence Dye Labeling and Tracking Analysis of PC-NVs

PC-NVs were labeled with red-fluorescent dye 1,1'-dioctadecyl-3,3,3',3'-tetramethylindodicarbocyanine, 4-chlorobenzenesulfonate salt (DiD) (Thermo Scientific, Fremont, CA, USA) according to the manufacturer's instructions. 2.5 µL of DiD dye solution was added to 100 µg of PC-NVs with total 500 µL of HBS. Incubated for 10 min at room temperature, diluted with 9.5 mL of cold HBS, and subjected to ultracentrifugation at 100,000 × g for 2 hours at 4°C. The pellets washed with HBS and re-centrifuged at 100,000 × g for 2 hours. DiD-labeled PC-NVs pellets then resuspended in HBS and used for tracking analysis. Penis tissues were harvested at indicated time point after injection and fixed with 4% formaldehyde for 24 hours at 4°C. Tissues were stained with antibodies and mounted with mounting medium containing the nuclear dye, 4',6-diamidino-2-phenylindole (DAPI; Vector Laboratories Inc., Burlingame, CA, USA). DiD dye tracking was performed using confocal fluorescence microscopy (K1-Fluo; Nanoscope Systems, Inc., Daejeon, Korea).

### Animal Treatment with PC-NVs

Eight-week-old male C57BL/6 mice were used in this study. DM was induced by intraperitoneal injections of streptozotocin (STZ; Sigma-Aldrich) in 0.1 M citrate buffer (pH 4.5) at a dose of 50 mg/kg body weight for 5 consecutive days.^51^ Tail vein blood glucose concentration greater than 300 mg/dL after 8 weeks after STZ injection confirmed DM. DM groups were injected with HBS (20 μl) or different concentration of PC-NVs (0.5, 1, or 5 µg in 20 μl of HBS) onto the penis on (days -3 and 0) while control group injected with same volume of HBS. Mice were anesthetized with ketamine (100 mg/kg) and xylazine (5 mg/kg) intraperitoneal. Placed supine on a thermoregulated surgical table with a vascular clamp placed on the base of the penis prior to injection. The clamp was left in place for 30 minutes to prevent blood backflow from the penis.

### Measurement of Erectile Function

Erectile function was measured according to protocols.^51^ Mice anesthetized with ketamine (100 mg/kg) and xylazine (5 mg/kg) by intramuscular injection. Cavernous nerve was stimulated with bipolar platinum wire electrodes placed under the cavernous nerve at 5 V/12 Hz and pulse width of 1 m/s. During tumescence, maximal intracavernous pressure (ICP) and area under the curve (total ICP) were recorded. Systemic blood pressure was measured with tail‐cuff blood pressure system (Visitech Systems, Apex, NC, USA). Systemic blood pressure variations were normalized by calculating ratios of the area under the curve (total ICP) or maximum ICP (cm H_2_O) to mean systolic blood pressure (MSBP).

### Diabetic Neurovasculature-mimicking *In Vitro* and *Ex Vivo* Experimental Systems

Diabetes-induced neuro-vasculopathy were mimicked *in vitro* by serum-starving cells for 24 hours then exposed to normal-glucose (NG, 5 mM glucose; Sigma-Aldrich) or high-glucose (HG, 30 mM glucose) conditions for 3 days (MCECs and MCPs) or 5 days (MPG and DRG tissue, and aortic ring) at 37°C in a humidified 5% CO_2_ atmosphere.^52^

### Tube-formation Assay

Tube-formation assays were performed based on protocols.^53^ Roughly 100 µL of growth factor-reduced Matrigel (BD Biosciences) was dispensed into each of 48-well culture plates at 4°C. Plates incubated at 37°C for at least 10 to 15 minutes, pretreated MCECs or MCPs were seeded onto the matrigel at 1 × 10^5^ cells/well in 300 µL of M199 or DMEM containing 2% FBS. Tube formation was observed for 18 hours under a phase-contrast microscope, and the amount of master junctions from seven separate experiments were determined in a blinded manner using Image J software (National Institutes of Health [NIH] 1.34, http://rsbweb.nih.gov/ij/).

### Aortic Ring Assay

Aortic rings were prepared from abdominal and thoracic aorta explants of 8-week-old C57BL/6 mice. Tissue positioned in wells of an 8-well Nunc Lab-Tek Slide System (Sigma-Aldrich). covered with 50 μl Matrigel. The aortas were cultured in complete M199 for 5 days in NG or HG medium with HBS or PC-NVs (1 μg/mL), or as indicated. Images of aortic segments and outgrowth were acquired with a phase-contrast microscope. The sprouting area was analyzed using Image J software (NIH 1.34, http://rsbweb.nih.gov/ij/).

### Cell-migration Assay

Pretreated MCECs were seeded onto the SPL Scar Block system (SPL Life Sciences, Pocheon-si, Gyeonggi-do, Korea) at >95% confluence in 60-mm culture dishes. The blocks were removed 5 hours after seeding, and cells were further incubated with M199 containing 2% FBS for 24 hours. Images were acquired with a phase-contrast microscope. Migrated cells were analyzed by measuring migrated cell proportion along the frame line.

### BrdU-incorporation assay

MCECs were treated with HBS or PC-NVs (1 µg/mL) under NG or HG conditions for 3 days, then treated with 10 µM BrdU (5'-bromo-2'-deoxyuridine (Sigma-Aldrich) at 37°C for 1 hour. The cells then fixed and stained with an anti-BrdU antibody (1:200; Bio-Rad, Hercules, CA, USA). The number of BrdU-positive endothelial cells was counted at a screen magnification of 400× and expressed per high-power field.

### TUNEL Assay

The MCECs cell death after treated with HBS or PC-NVs (1 µg/mL) under NG or HG conditions were evaluated by TUNEL (terminal deoxynucleotidyl transferase-mediated deoxyuridine triphosphate nick-end labeling) assay using the ApopTag Fluoresce In Situ Apoptosis Detection Kit (Chemicon, Temecula, CA, USA) according to the manufacturer's instructions. Numbers of apoptotic cells were determined at a 200× magnification by confocal fluorescence microscope and expressed per high-power field.

### *Ex Vivo* Neurite Sprouting Assay

Mouse MPG and DRG tissues were prepared and maintained as described previously.^54^ MPG tissues closely attached to the ventral part of the prostate and DRG tissues from L3-L5 were isolated from 8-week-old C57BL/6 male mice under a dissecting microscope, transferred into sterile vials containing Hank's balanced salt solution (HBSS; Gibco), and then rinsed and washed twice with PBS. MPG and DRG tissues were cut into small pieces and plated on poly-D-lysine hydrobromide-coated, 8-well Nunc Lab-Tek Chamber Slides (Sigma-Aldrich), then entirely covered with Matrigel and incubated for 10-15 minutes at 37°C in a 5% CO_2_ environment. Thereafter, 1 mL of complete Neurobasal medium (Gibco) supplemented with 2% serum-free B-27 (Gibco) and 0.5 nM GlutaMAX-I (Gibco) were added and MPG and DRG tissues were incubated under NG or HG conditions with HBS, PC-NVs (1 μg/mL), or other conditions as described in the text. Five days later, neurite outgrowth was assessed by fixing tissue segments in 4% paraformaldehyde for at least 30 minutes and immunostaining with an anti-βIII tubulin antibody (1:100; Abcam, Cambridge, MA, USA).

### RNA Sequencing Analysis

For RNA-sequencing analysis, MCECs were cultured and treated with HBS or PC-NVs (1 μg/mL) under NG or HG conditions. RNA-sequencing analyses were performed by E-Biogen Inc. (Seoul, Korea) as described previously.^8^. Total RNA isolated 72 hours after exposure to glucose conditions and 24 hours after exposure to PC-NVs using TRIzol reagent (Invitrogen). RNA quality was assessed using an Agilent 2100 Bioanalyzer (Agilent Technologies, Amstelveen, The Netherlands). RNA was quantified using ND-2000 Spectrophotometer (Thermo Inc., Wilmington, DE, USA).

### Library Sequencing and Data Analysis

Libraries were prepared from total RNA using a SMARTer Stranded RNA-Seq Kit (Clontech Laboratories, Inc., USA). mRNA was isolated with Poly (A) RNA Selection Kit (LEXOGEN, Inc., Austria); indexed with Illumina indices 1-12; and enriched with PCR. Libraries were assessed for mean fragment size using an Agilent 2100 Bioanalyzer (DNA High Sensitivity Kit), quantified with StepOne Real-Time PCR System (Life Technologies, Inc., USA). High-throughput sequencing was made as paired-end 100 sequencing with HiSeq 2500 system (Illumina, Inc., USA). FastQC (https://www.bioinformatics.babraham.ac.uk/projects/fastqc/) for quality control of raw sequencing data. Adapters, and low-quality reads (<Q20) were removed using FASTX_Trimmer (http://hannonlab.cshl.edu/fastx_toolkit/) and BBMap (https://sourceforge.net/projects/bbmap/), trimmed reads were mapped to reference with TopHat.^55^ Gene expression levels were estimated based on RC (read count) and FPKM (fragments per kb per million reads) values determined using BEDTools^56^ and Cufflinks.^57^ then normalized with EdgeR within R (https://www.r-project.org) Quantile normalization. Data mining and graphic visualized with ExDEGA (E-Biogen, Inc., Seoul, Republic of Korea). RNA-sequencing data have been stored at Gene Expression Omnibus database (www.ncbi.nlm.nih.gov/geo accession no. GSE183220).

### Lentiviral-mediated shRNA Delivery

For lentiviral infection of MCPs, SMART vector mouse lentivirus containing shRNA targeting Lcn2 (shLCN2; Dharmacon, Lafayette, CO, USA) or scrambled control shRNA (shCon) lentiviruses (Santa Cruz Biotechnology Inc, Dallas, TX USA) were added to culture medium at a concentration of 5 × 10^4^ TU/mL. The sequence of the shRNA targeting mouse Lcn2 was 5'‑ACAGAAGGCAGCTTTACGA-3'. Experiments were performed 3 days after lentivirus infection.

### Signaling Explorer Antibody Arrays

Mouse cavernosum tissues from age-matched control and diabetic mice intracavernous injected with HBS (H), PC-NVs, or Lcn2-knockdown (KD) PC-NVs were used for Signaling Explorer Antibody Array (Fullmoon Biosystems, Sunnyvale, CA, USA), performed according to the manufacturer's instructions. Assay consists of 1358 unique antibodies covering 20 cell-signaling pathways, was performed as a custom service by E-Biogen (Ebiogen Inc.).

### Clustering and Protein Interaction Network Analysis

Hierarchical clustering analyses were performed with ExDEGA Graphic plus program (Ebiogen, Inc.) based on Euclidean distance correlation measurement with average linkage.^58^ Clusters and heat maps were visualized with the ExDEGA Graphic Plus program. Networks were visualized and analyzed with STRING (Search Tool for the Retrieval of Interacting Genes/Proteins; https://string-db.org), and scored according to their confidence.^38^

### Histological Examinations

Explants for immunofluorescence (penis, MPG, and DRG tissues) were fixed in 4% paraformaldehyde for 24 hours at 4°C. Frozen tissue sections (15-µm thick) or *ex vivo* samples were incubated at 4°C overnight with indicated primary antibodies against the following proteins: smooth muscle α-actin (α-SMA, 1:100; Sigma-Aldrich), neuron-glial antigen 2 (NG2, 1:100; Millipore, Temecula, CA, USA), platelet/endothelial adhesion molecule 1 (PECAM-1, 1:100; Millipore), phosphorylated eNOS (p-eNOS, 1:100; Cell Signaling, Beverly, MA, USA), neurofilaments (NF, 1:100; Sigma-Aldrich), neuronal NOS (n-NOS, 1:100; Santa Cruz Biotechnology Inc, Dallas, TX, USA), claudin-5 (1:100; Invitrogen, Carlsbad, CA, USA), occludin (1:100; Novus Biologicals), βIII tubulin (1:100; Abcam), or oxidized low-density lipoprotein (Ox-LDL, 1:100; Abcam). After several washes with PBS, samples were incubated with species-appropriate tetramethyl rhodamine isothiocyanate (TRITC)- or fluorescein isothiocyanate (FITC)-conjugated secondary antibodies (1:100; Zymed Laboratories, South San Francisco, CA, USA) for 2 hours at room temperature and mounted with nuclear dye DAPI (Vector Laboratories, Inc., Burlingame, CA, USA) contained solution. Fluorescence signals were visualized using confocal microscope (K1-Fluo; Nanoscope Systems, Inc.). Quantitative analyses were performed using Image J software (NIH 1.34, http://rsbweb.nih.gov/ij/).

### Western Blotting

Penis tissues were lysed in RIPA buffer (Sigma-Aldrich) with protease inhibitors (1:100; Sigma-Aldrich) and phosphatase inhibitors (1:100; Sigma-Aldrich). Equal amounts of protein (40 µg per lane) electrophoresed on SDS-PAGE gels (8% to 15%), transferred to polyvinylidene difluoride membranes, blocked with 5% nonfat dry milk (BD Biosciences) for 1 hour at room temperature. Membranes were probed with antibodies to eNOS (1:500, Cell Signaling), p-eNOS (1:500; Cell Signaling), neurotrophin-3 (NT-3, 1:500; Santa Cruz Biotechnology Inc, Dallas, TX, USA), brain-derived neurotrophic factor (BDNF, 1:500; Santa Cruz Biotechnology Inc), nerve growth factor (NGF, 1:500; Santa Cruz Biotechnology Inc), Lcn2 (1:1000; Abcam), MAPK (1:1000, Cell Signaling), p-MAPK (1:1000; Cell Signaling), PI3K (1:1000; Cell Signaling), p-PI3K (1:1000, Cell Signaling), AKT (1:1000, Cell Signaling), p-AKT (1:1000, Cell Signaling), Trp53 (1:1000, Abcam), p-Trp53 (1:1000; Cell Signaling), or β-actin (1:5000; Cell Signaling). The results were quantified by densitometry using Image J software (NIH 1.34, http://rsbweb.nih.gov/ij/).

### Statistical Analysis

Data are expressed as means ± SEM from five independent experiments. For parametric data, intergroup assessments made with one-way analysis of variance (ANOVA) followed by Newman-Keuls posthoc tests. Kruskal-Wallis's test was used nonparametric data. Probability value analysis was performed using Graph Pad Prism software. P-values < 0.05 were considered statistically significant; individual P-values are indicated in figure legends (*P< 0.05, ^#^*P*< 0.001).

## Supplementary Material

Supplementary figures and tables.
