# Supplementary material for: Pericyte-derived extracellular vesicle-mimetic nanovesicles ameliorate erectile dysfunction via lipocalin 2 in diabetic mice
Source: Int J Biol Sci. 2022 May 16;18(9):3653–67. doi: 10.7150/ijbs.72243 (PMC9254477; doi:10.7150/ijbs.72243)
Supplement: Supplementary file 1 — Supplementary figures and tables. [file ijbsv18p3653s1.pdf]

Supplemental Information

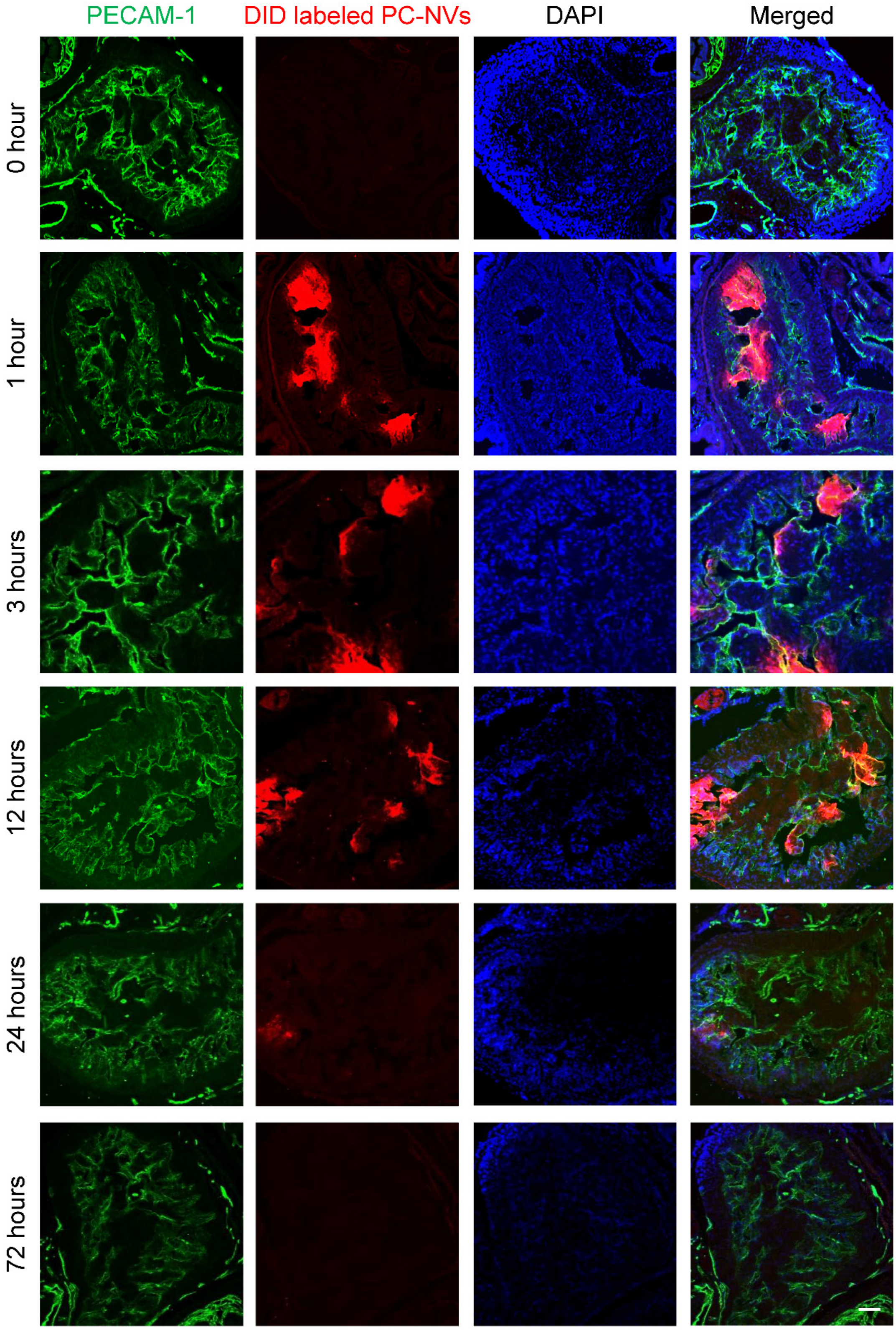

Figure S1: In vivo detection of DiD-red fluorescently labeled pericyte (PC)-derived extracellular vesicle-mimetic nanovesicles (PC-NVs) in the penis of normal mice. The penis tissue was harvested 0, 1, 3, 12, 24, and 72 hours after intracavernous injection of DiD-red labeled PC-NVs into the normal mice. Representative immunohistochemical staining with PECAM-1 (green) in cavernous tissue from normal mice. Nuclei were labeled with the DNA dye DAPI. Scale bar = 200 µm. DAPI = 4,6-diamidino-2-phenylindole.

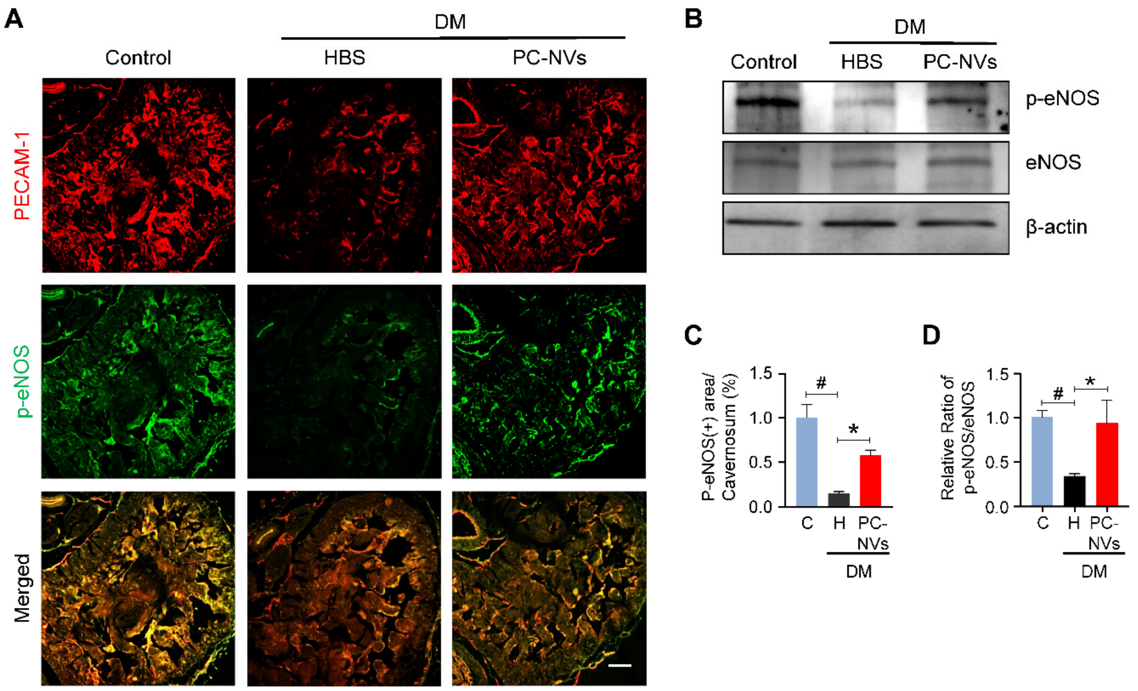

Figure S2: PC-NVs induce cavernous eNOS phosphorylation (p-eNOS) in diabetic mice. (A) PECAM-1 (red) and phosphor-eNOS (green) staining of cavernous tissue from age-matched control (C) and diabetic mice obtained 2 weeks after intracavernous injection of HBS (H) or PC-NVs (5 µg/20 µl) on days −3 and 0. Scale bar = 100 µm. (B) Representative western blots for phosphor-eNOS and eNOS in age-matched control (c) and diabetic mice obtained 2 weeks after intracavernous injection of HBS (H) or PC-NVs (5 µg/20 µl) on days −3 and 0.

(C) Quantification of the phosphor-eNOS immunopositive area in cavernous tissue by Image J (n = 8). (D) Band intensity values of indicated proteins was quantified by assessing the density of the corresponding protein bands using Image J (n = 4). Results are presented as means ± SEM (*$P < 0.05$; $^{\#}P < 0.001$). The relative ratio in the control group was set to 1. DM, diabetes mellitus; HBS, HEPES-buffered saline.

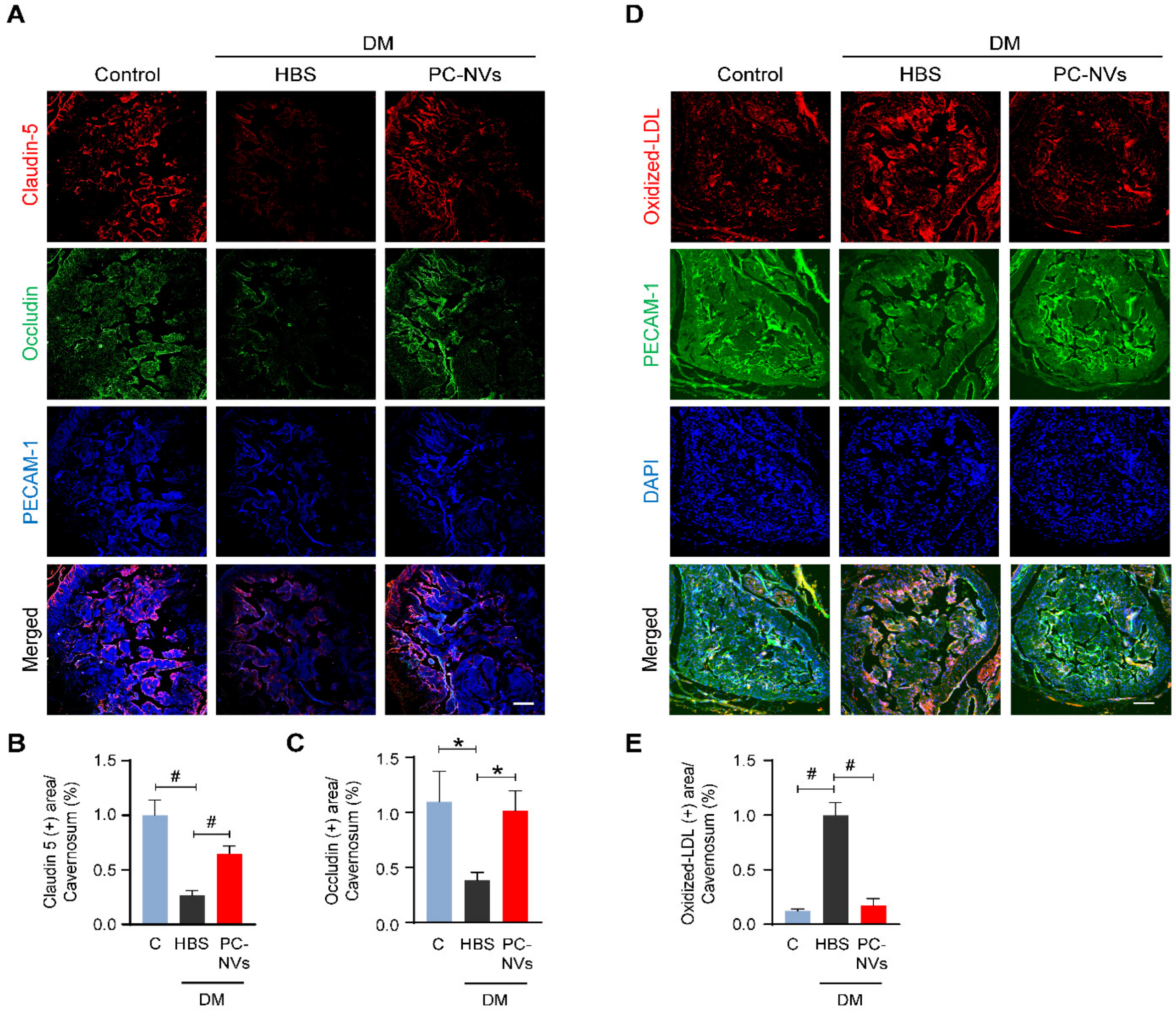

Figure S3: PC-NVs preserve endothelial cell-cell junctions and decrease the extravasation of oxidized-LDL under diabetic conditions. (A-C) Triple-immunostaining for Claudin-5 (red), Occludin (green), and PECAM-1 (blue) in age-matched control (C) and diabetic mice obtained 2

weeks after intracavernous injection of HBS (H) or PC-NVs (5 μg/20 μl) on days −3 and 0. Scale bar = 100 μm. Quantification of claudin-5- and occluding-immunopositive area in the cavernosum using Image J (n = 10). Results are presented as means ± SEM (*$P < 0.05$; $^{\#}P < 0.001$). (D and E) PECAM-1 (green) and oxidized-LDL (red) staining of cavernous tissue from age-matched control (C) and diabetic mice obtained 2 weeks after intracavernous injection of HBS (H) or PC-NVs (5 μg/20 μl) on days −3 and 0. Scale bar = 100 μm. Quantification of oxidized-LDL-immunopositive area in the cavernosum using Image J (n = 7). Results are presented as means ± SEM ($^{\#}P < 0.001$). DM, diabetes mellitus; HBS, HEPES-buffered saline.

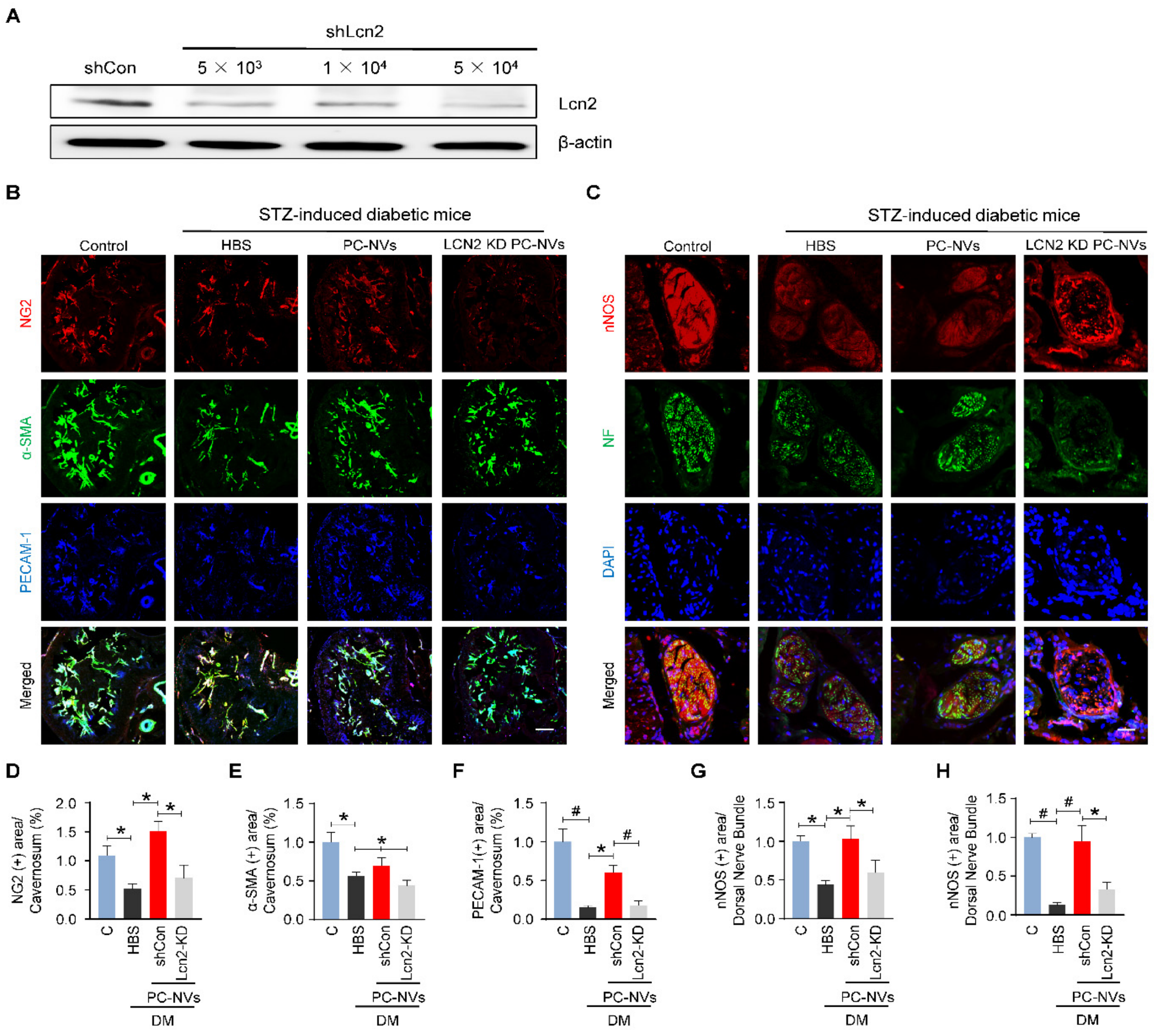

Figure S4: PC-NVs induce neurovascular regeneration with Lcn2-dependent manner under diabetic conditions. (A) Representative Western blots for Lcn2 in mouse cavernous pericyte infected with shCon or shLcn2 lentivirus at three doses (5×10^3 TU, 1×10^4 TU, and 5×10^4 TU/ml culture medium) for at least 3 days. (B) Triple-immunostaining for NG2 (red), α-SMA (green), and PECAM-1 (blue) in age-matched control (C) and diabetic mice obtained 2 weeks after intracavernous injection of HBS (H), PC-NVs (shCon) (5 μg/20 μl), or PC-NVs (Lcn2-KD) (5 μg/20 μl) on days −3 and 0. Scale bar = 100 μm. (C) nNOS (red) and neurofilament (NF, green) staining of cavernous tissue in age-matched control (C) and diabetic mice obtained 2 weeks after intracavernous injection of HBS (H), PC-NVs (shCon) (5 μg/20 μl), or PC-NVs (Lcn2-KD) (5 μg/20 μl) on days −3 and 0. Scale bar = 25 μm. (D- H) Intensity quantification of each protein-immunopositive area in the cavernosum using Image J (n = 5). Results are presented as means ± SEM ($^{*}P < 0.05$; $^{\#}P < 0.001$). DM, diabetes mellitus; HBS, HEPES-buffered saline.

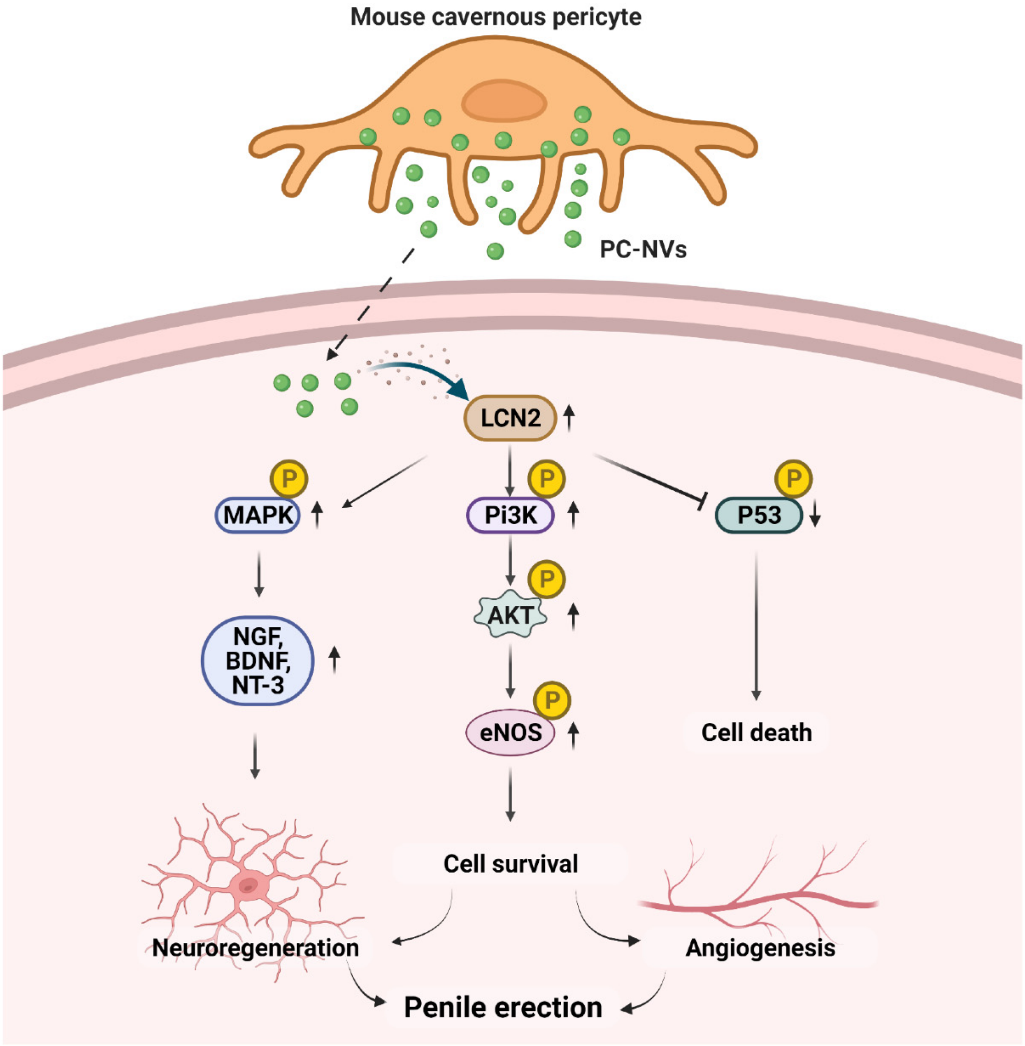

Figure S5: Schematic depiction of the proposed mechanism for Lcn2-dependent restoration of diabetic ED by PC-NVs. PC-NVs, Pericyte-derived extracellular vesicle-mimetic nanovesicles; Lcn2, lipocalin 2; PI3K, phosphoinositide 3; Akt, protein kinase B; eNOS, endothelial nitric oxide synthase; MAPK, mitogen-activated protein kinase; NGF, nerve growth factor; BDNF, brain-derived neurotrophic factor; NT-3, neurotrophin-3; P53, tumor protein 53.

Table S1. The summary of Pericyte derived PC-NVs characteristics

**Comparator**	Cell or materials	Preparation strategy	Particle Characterization	Comparison to natural exosome	Potential application
**EV-mimetic NVs**	Mouse Cavernous Pericytes	serial extrusion (10 µm, 5 µm, 1 µm) + OptiPrep DGUC	Size by TEM and Zeta potential: 134.5±16.92 nm	Similar cup like morphology, size distribution, protein markers	siRNA delivery, cell proliferation enhancement, pro-angio-neurogenesis, nanocarrier alternative to exosome,

DGUC; density gradient ultra-centrifugation, TEM; transmission electron microscopy.

Table S2. Physiologic and metabolic parameters: 2 weeks after treatment with different doses of PC-NVs

		STZ-induced diabetic mice			
	Control	HBS	PC-NVs 0.5 µg	PC-NVs 1.0 µg	PC-NVs 5.0 µg
Body weight (g)	29.1 ± 0.3	22.8 ± 1.1#	23.0 ± 0.8#	22.6 ± 0.4#	22.4 ± 0.2#
Postprandial glucose (mg/dL)	148.3 ± 7.2	577.4 ± 9#	575.3 ± 12.6#	572.7 ± 9#	571.3 ± 13.5#
Fasting glucose (mg/dL)	113.9 ± 5.7	416.4 ± 8.8#	411.0 ± 8.5#	408 ± 19.5#	393.4 ± 19.7#
MSBP (cm H2O)	83 ± 3.1	89.9 ± 1.1	87.8 ± 5.1	71.33 ± 0.7	73.89 ± 1.6

Values are the mean ± SEM for n = 5 animals per group. #*P*< 0.001 vs. control group. MSBP, mean systolic blood pressure; STZ, streptozotocin.

Table S3. Physiologic and metabolic parameters: 2 weeks after treatment with PC-NVs (shCon) and PC-NVs (Lcn2-KD) under diabetic conditions.

		STZ-induced diabetic mice		
	Control	HBS	PC-NVs (shCon)	PC-NVs (Lcn2-KD)
Body weight (g)	28.5 ± 0.6	24.9 ± 1.4#	24.3 ± 1.6#	22.2 ± 1.2#
Postprandial glucose (mg/dL)	123.6 ± 2.6	550.0 ± 21.7#	489.4 ± 19.5#	517.4 ± 22.15#
Fasting glucose (mg/dL)	100 ± 3.8	376.1 ± 22.3#	347.6 ± 18.4#	422.0 ± 23.9#
MSBP (cm H2O)	90.1 ± 1.1	94.3 ± 2.2	98.9 ± 4.1	104.4 ± 2.1

Values are the mean ± SEM for n = 5 animals per group. #*P*< 0.001 vs. control group. MSBP, mean systolic blood pressure; STZ, streptozotocin.
